# Intra- and Inter-Rater Reliability of Processing Ultrasound Tissue Characterization Scans in Midportion Achilles Tendinopathy

**DOI:** 10.1155/2022/9348298

**Published:** 2022-06-16

**Authors:** Marc Adriaan Paantjens, Pieter Herman Helmhout, Franciscus Jacobus Gerardus Backx, Meindert Thomas Annechien Willibrord Martens, Joeri Paulus Adrianus van Dongen, Eric Wilhelmus Petrus Bakker

**Affiliations:** ^1^Sports Medicine Centre, Training Medicine and Training Physiology, Royal Netherlands Army, Utrecht, Netherlands; ^2^Department of Rehabilitation, Physical Therapy Science and Sports, University Medical Center Utrecht, Utrecht, Netherlands; ^3^Centre of Excellence, Training Medicine and Training Physiology, Royal Netherlands Army, Utrecht, Netherlands; ^4^Fontys University of Applied Science, Eindhoven, Netherlands; ^5^National Institute of Musculoskeletal Ultrasound, Vianen, Netherlands; ^6^Department Epidemiology and Data Science, Division EPM, University Medical Center Amsterdam, Amsterdam, Netherlands

## Abstract

**Purpose:**

Ultrasound tissue characterization (UTC) is used to visualize and quantify the Achilles tendon structure. We investigated the intra-rater and inter-rater reliability of UTC for quantifying the midportion tendon structure and the area of maximum degeneration (AoMD) in military personnel with midportion Achilles tendinopathy.

**Method:**

UTC scans of 50 patients (16–60 years) were processed twice by rater 1 and once by rater 2. First, the midportion tendon structure was quantified and subsequently the AoMD. The intraclass correlation coefficient (ICC) was calculated for echo-types I, II, III, IV, aligned fibrillar structure (echo-types I + II), and disorganized tendon structure (echo-types III + IV).

**Results:**

For midportion tendon structure, all ICC values were excellent for intra-rater reliability (range: 0.97 to 0.99) and inter-rater reliability (range: 0.98 to 0.99). Regarding the AoMD, intra-rater reliability showed excellent ICC values for all echo-types (range: 0.94 to 0.98) except for echo-type II (0.85). Inter-rater reliability showed excellent ICC values for all echo-types (range: 0.92 to 0.98).

**Conclusion:**

Processing of UTC scans is highly reliable in quantifying the midportion Achilles tendon structure and the AoMD.

## 1. Introduction

Achilles tendinopathy (AT) is a painful overuse condition [1] that affects the tendon midportion more frequently (55–65%) than the insertion (20–25%) [2], specifically between the ages of 30 and 50 years [3]. In recreational running, about one in 20 runners develops AT [4], and one in three runners experiences persisting symptoms 1 year after new-onset AT [5]. AT is also common in the military [6] where musculoskeletal injuries may affect combat readiness [7] and can result in discharge [8].

Histologically, AT is considered a degenerative condition, in which the pathogenesis may be regarded as a continuum [9,10]. As degeneration progresses, the tendon's ability to regain normal morphology and architecture is considered to decrease [9,10]. Approximately 4% of all patients that were previously diagnosed with AT sustain an Achilles tendon rupture [11]. While spontaneous tendon rupturing is, almost without exception, preceded by degenerative changes [12], the extent of degeneration in ruptured Achilles tendons appears more severe than in tendinopathic Achilles tendons [13].

Ultrasound tissue characterization (UTC) is a noninvasive imaging modality that is reported to be able to visualize the Achilles tendon structure and quantify the Achilles tendon matrix integrity [14]. In AT, UTC is used to evaluate nonsurgical [15, 16] and surgical interventions [17, 18] targeting the Achilles tendon structure. UTC can also be used to monitor the reaction of the tendon to load, [19, 20] as this is considered of importance in preventing progression of tendon degeneration in AT. [9, 10].

While the scanning procedure in UTC is relatively standardized and automated, the processing of UTC scans to quantify the Achilles tendon structure is highly operator dependent. Processing is performed manually, and depends on the assessor's ability to mark the anatomical borders of the Achilles tendon in the anatomical region of interest, in consecutive, short-axis images. The processing of UTC scans has been tested for reliability in patellar tendons, [21] but not yet in Achilles tendons. Our objective was to determine the intra-rater and inter-rater reliability of processing UTC scans in a large cohort of military personnel suffering from midportion Achilles tendinopathy (mid-AT).

## 2. Materials and Methods

### 2.1. Study Setting and Participants

The study was conducted at the Department of Sports Medicine of the Royal Netherlands Army, Utrecht, the Netherlands. The UTC scans had been collected as part of an observational study (https://www.toetsingonline.nl/to/ccmo_search.nsf/Searchform?OpenForm, Dossier number ToetsingOnline: NL69527.028.19) aiming to evaluate shockwave therapy, load management, and return to running as standard care for mid-AT.

### 2.2. Enrollment

Consecutive patients consulting the Department of Sports Medicine between February 2019 and January 2021 were eligible if the following inclusion criteria were met: (1) military personnel in active duty (18–60 years), (2) patients with a clinically established diagnosis of mid-AT [22], and (3) symptoms for two months or more. In case of bilateral symptoms, only the most severely affected side, defined as the side with the lowest score on the Victorian Institute of Sports Assessment-Achilles (VISA-A) questionnaire, was included in the analysis. Subjects were excluded if they reported (1) concomitant insertional Achilles tendinopathy (ins-AT) and (2) factors that are known to adversely affect the Achilles tendon morphology and architecture, i.e., signs of a complete Achilles tendon rupture; prior surgery to the Achilles tendon; use of statins, fluoroquinolones, or corticosteroids; [23,24] and a previous diagnosis of rheumatoid arthritis, diabetes mellitus, or psoriasis [25]. All participants were recruited by the main researcher (MP, physical therapist).

### 2.3. Ethical Considerations

The study protocol was reviewed by the ethics committee METC Brabant, Tilburg, the Netherlands (NW2021-69), and was judged not to be subjected to the Medical Research Involving Human Subjects Act. All participants provided written informed consent for anonymous use of their data.

### 2.4. Patient Characteristics

Patient characteristics and UTC scans were collected by the main researcher, who had 8 years of experience in UTC. The following characteristics were retrieved: age (years), height (cm), weight (kg), gender (male/female), body mass index (BMI, in%), symptom duration (months), baseline VISA-A scores, and baseline numeric rating scale (NRS) scores for maximum Achilles tendon pain.

The VISA-A is considered the gold standard for assessing pain and function in AT, ranging from 0 to 100 points, where 100 represents a perfect asymptomatic score [22, 26]. While the validity and reliability of the NRS for pain (ranging from 0, no pain, to 10, worst conceivable pain) has not yet been formally established in AT, it is often used to evaluate progress in these patients [26]. The NRS has been proven valid in numerous musculoskeletal pain conditions [26].

### 2.5. UTC Scanning

**Figure 1:**
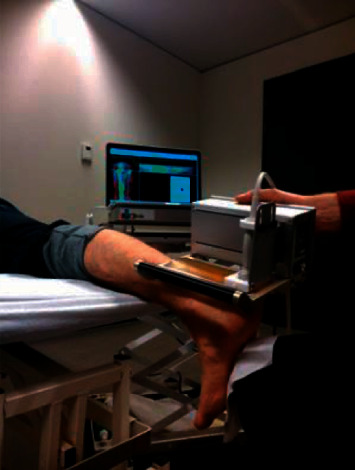
UTC-scanning procedure.

All UTC scans were performed (Figure 1) using a standardized scanning protocol [18, 27] as well as standardized ultrasound parameters (12 MHz, depth: 3 cm, focus: 1.3 cm). Prior to scanning, all patients were asked to lay prone with their feet hanging freely over the examining table. The main researcher sat on a stool behind the treatment couch, and used his knee to fix the patients ipsilateral foot in maximum dorsiflexion. In this way, perpendicular scanning was secured over the length of the Achilles tendon. A UTC tracker (UTC Imaging, 6171 GD Stein, the Netherlands, serial no. UTC-201-041) was placed over the Achilles tendon and remained in a manually fixed position during the scanning procedure.

A 12-MHz linear-array transducer (Terason 12L5 SmartProbe, Vermon, France) using Terason software (T2000 + OEM) was embedded in the UTC tracker, ensuring a fixed angle of insonation. The transducer was mounted to move automatically over an acoustic standoff pad. Ultrasound transmission gel (Aquasonic 100, Hannover, Germany) was applied between the transducer and the acoustic standoff and also on the Achilles tendon.

**Figure 2:**
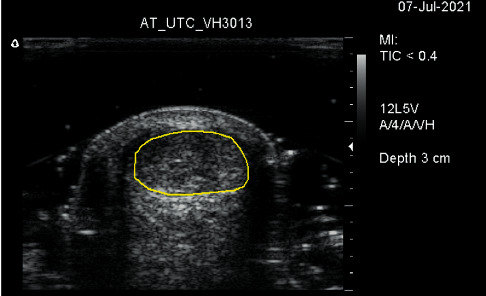
Short-axis grayscale image of the midportion of the Achilles tendon. The yellow circle marks the anatomical borders of the Achilles tendon.

During UTC scanning, a motor drive automatically moved the transducer 12 centimeter forth and back over the Achilles tendon, capturing a short-axis grayscale image (Figure 2) every 0.2 millimeters. The total time of the scanning procedure is less than 45 seconds. The images were stored on a computer and a backup was created.

**Figure 3:**
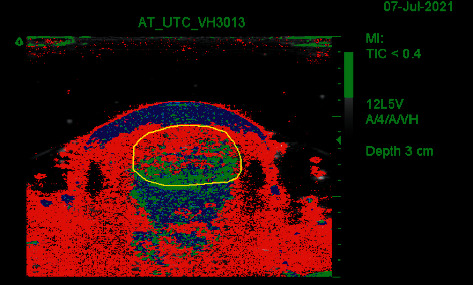
Short-axis UTC image of the midportion of the Achilles tendon. The yellow circle marks the anatomical borders of the Achilles tendon. Echo-type I is colored green, echo-type II is colored blue, echo-type III is colored red, and echo-type IV is colored black. Echo-types I + II represent aligned fibrillar structure whereas echo-types III + IV can be seen as disorganized Achilles tendon structure.

**Figure 4:**
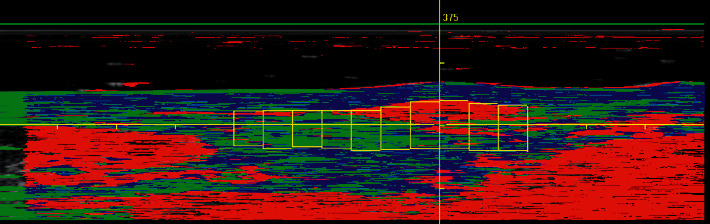
Long-axis UTC image of the Achilles tendon. Eleven consecutive contours (vertical yellow lines) capture the entire tendon midportion, starting 2 cm proximal to the calcaneus (most right sided contour) and continuing up to the myotendinous junction (most left sided contour). Echo-type I is colored green, echo-type II is colored blue, echo-type III is colored red, and echo-type IV is colored black. Echo-types I + II represent aligned fibrillar structure whereas echo-types III + IV can be seen as disorganized Achilles tendon structure.

Subsequently, the grayscale images were grouped into a three-dimensional volume block of data, allowing tomographic visualization of the Achilles tendon in three planes: coronal, transverse, and sagittal. A validated algorithm analyzed the three-dimensional stability of the grayscale echo patterns, quantifying the Achilles tendon structure in percentages of echo-type I (colored green), echo-type II (colored blue), echo-type III (colored red), and echo-type IV (colored black) (Figures 3 and 4) [14]. Research has shown that grayscale dynamics are strongly related to tendon architecture and histopathology [28]. Echo-type I is the most stable echo pattern of consecutive short-axis images, while echo-type IV is the least stable echo pattern. Together, echo-types I + II are considered to represent aligned fibrillar structure, whereas echo-types III + IV can be seen as disorganized Achilles tendon structure. Aligned fibrillar structure and disorganized tendon structure can be used as outcome measures when evaluating patients with UTC [14,16–18,21]. Normative data for the Achilles tendon structure in asymptomatic individuals, with regard to age, race, and gender, has recently been published [29].

All participants were instructed not to engage in any sports activities involving running and jumping for at least 48-hours prior to UTC scanning, in order to exclude possible transient load-related changes in UTC echo pattern [19].

### 2.6. Intra-Rater Reliability and Inter-Rater Reliability

Two physical therapists (MSc) performed all measurements for the study. Rater 1 (MM) had 9 years of experience in musculoskeletal sonography, and rater 2 (JvD) had 8 years of experience.

Prior to the study, both raters, who were unfamiliar with UTC, participated in a 3-day consensus procedure, consisting of instruction and practice, to standardize the processing of the UTC scans. During this procedure, each rater processed 10 UTC scans.

All UTC scans were collected by the main researcher (MP) and were anonymized and processed independently by these two raters. Rater 1 processed each UTC scan twice with at least four weeks in between to determine the intra-rater reliability.

### 2.7. Processing of UTC Scans

**Figure 5:**
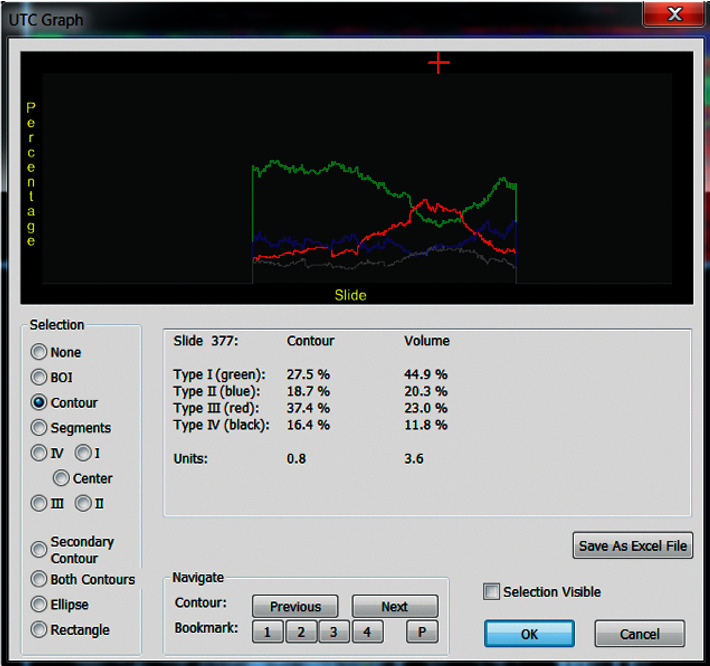
UTC graph of the midportion Achilles tendon structure. The red cross indicates from which short-axis image the figures under *contour* (=AoMD) come from. This position can be changed by sliding through the sagittal plane. A certain plane is identified by the number mentioned by *slide*. In this graph, the AoMD consists of 27.5% echo-type I while the total *volume* of the Achilles tendon midportion (between the first and last contour) consists of 44.9% echo-type I.

First, both raters quantified the structure of the Achilles tendon midportion, defined as the part of the Achilles tendon 2–7 cm proximal to the calcaneal insertion [22]. For this, each rater marked the contours of the Achilles tendon borders in consecutive short-axis images (Figures 2 and 3) using UTC software. The first contour was placed 2 centimeters (101 frames) proximal to the calcaneus, continuing every 0.5 cm (25 frames) up to the myotendinous junction or a length of 7 cm (maximum of 11 contours) (Figures 2–4). The contours were automatically interpolated, and the tendon volume between the first and the last contour was expressed in percentages of echo-types I, II, III, and IV (Figure 5). Both raters used the default setting of window size 25 (the stability of the grayscale echo pattern over 4.8 millimeters) for contour marking, and the default setting of window size 17 (the stability of the grayscale echo pattern over 3.2 millimeters) for the quantification of tendon structure. All contours were saved to the corresponding UTC images.

Following structural quantification of the Achilles tendon midportion, both raters identified the area of maximum degeneration (AoMD) in the tendon midportion (Figure 5). The AoMD was defined as the area (1 frame) in which intact and aligned tendon bundles (echo-type I) were lowest represented. The AoMD was identified by using a slider in the UTC graph while reading the values of echo-type I in real time (Figure 5).

Data were extracted using a standardized Microsoft Excel extract form.

For this reliability study, we recruited a sample of 50 patients (50 symptomatic midportion Achilles tendons) [30].

### 2.8. Statistical Analysis

Baseline characteristics of our studied population were presented with appropriate measures of central tendency and dispersion. The intraclass correlation coefficient (ICC) was used to assess the intra-rater and the inter-rater reliability. The ICC was calculated (ICC: 2.1, two-way random, single measurement, absolute agreement) for echo-types I, II, III, IV, aligned fibrillar structure (echo-types I + II), and disorganized tendon structure (echo-types III + IV). For the interpretation of the ICC, we adopted the guideline of Koo and Li [31] in which values were considered to represent poor (ICC <0.5), moderate (ICC = 0.5–0.75), good (ICC = 0.75–0.90), and excellent (ICC >0.90) reliability. We also calculated the standard error of measurement $\text{SEM}=\text{SD of population}\times \sqrt{\left(1-\text{ICC}\right)}$) and the minimal detectable change $\left(\text{MDC}=1\text{.}96\times \text{SEM}\times \sqrt{2}\right)$. MDC values can be used to distinguish true differences in Achilles tendon matrix integrity from random variation.

All analyses were performed using SPSS (IBM SPSS Statistics for Windows, Version 25.0, IBM Corp., Armonk, NY).

## 3. Results

From March to July 2021, all UTC scans were independently processed by the two raters to ensure blinding of the procedure. A total of 50 scans of service members with symptomatic mid-AT were included in the study. Both raters indicated that all UTC scans were of sufficient quality to perform the rating procedure. Bilateral symptoms were present in 14 out of 50 participants (28%). In these cases, we only included the side with the lowest VISA-A score in the analysis. Patient characteristics are presented in Table 1. The mean echo-type percentages of the study population are displayed in Table 2.

For quantification of the structure of the Achilles tendon midportion, the ICC showed overall excellent scores for intra-rater reliability ranging from 0.97 (0.94–0.99) to 0.99 (0.97–0.99), as well as for inter-rater reliability ranging from 0.98 (0.95–0.99) to 0.99 (0.99–1.00) (Table 3). MDC values for intra-rater and inter-rater reliability ranged from 1.9% to 5.9% and 1.9% to 6.0%, respectively (Table 3).

Quantification of the AoMD also resulted in excellent ICC values for intra-rater reliability, ranging from 0.94 (0.90–0.97) to 0.98 (0.97–0.99), except for echo-type II 0.85 (0.75–0.91) which was considered good reliability (Table 4). ICCs for inter-rater reliability in assessing the AoMD were all excellent, ranging from 0.92 (0.86–0.95) to 0.98 (0.95–0.99).

MDC values for intra-rater reliability ranged from 4.6% to 10.0%, and for inter-rater reliability from 3.3 to 8.6% (Table 4).

## 4. Discussion

This is the first study to investigate both the intra-rater and inter-rater reliability for the processing of UTC scans in patients suffering from mid-AT. Excellent ICCs were found for the processing of the midportion Achilles tendon structure, ranging from 0.97 to 0.99 (intra-rater reliability) and from 0.98 to 0.99 (inter-rater reliability) (Table 3). Furthermore, for the quantification of the AoMD, we found excellent ICC values for intra-rater and inter-rater reliability (ICC all ≥0.92), except for the intra-rater reliability for echo-type II (ICC 0.85), which was considered good reliability (Table 4). We cannot explain this outlier.

To our current knowledge, quantifying the AoMD has not been previously reported, or tested for intra-rater and inter-rater reliability; therefore, we cannot compare these results to the literature.

Our results for the processing of the midportion Achilles tendon structure are comparable to those reported for patellar tendon structure, as van Ark et al. [21] reported similar ICCs for intra-rater reliability (ICC 0.97 to 0.99) and slightly lower ICCs for inter-rater reliability (ICC 0.84 to 0.94). The lower ICCs for inter-rater reliability found in their study may be attributed either to artifacts caused by the near presence of the apex patella, or to the fact that the optimal knee angle to perform a UTC scan can vary, which makes standardization difficult, in contrast to the ankle joint [21].

The inter-rater reliability of UTC for the midportion Achilles tendon structure was previously investigated in a study [14] where two raters individually collected and processed UTC scans. In this study, van Schie et al. [14] reported slightly lower ICCs compared to our study, ranging from 0.92 to 0.95. The fact that these raters analyzed different UTC scans, along with the use of a not yet automated scanning procedure, may have accounted for their somewhat lower inter-rater reliability scores. Moreover, UTC equipment advanced in the last decade, incorporating a higher frequency transducer, most likely resulting in superior imaging quality, thus making it easier for raters to distinguish the Achilles tendon borders from the peritendinous structures. Additional methodological differences between the two studies may hamper a direct comparison between the reported ICCs: van Schie et al. [14] quantified a relatively small region of interest in the Achilles tendon midportion (4 mm, 3 contours), in a mixed cohort of symptomatic and asymptomatic individuals, whereas in our study, the full anatomical midportion of the Achilles tendon (up to 5 cm or a maximum of 11 contours) was analyzed, including only symptomatic participants. Despite all these differences, outcomes were quite comparable between the two studies. In other words, including a nonautomated scanning procedure with a lower frequency transducer into the reliability analysis, analyzing different regions of interest, and targeting either symptomatic or asymptomatic cohorts all appear to have little influence on the high inter-rater reliability of UTC.

The intra-rater reliability of UTC for Achilles tendon structure was previously tested in a smaller cohort (*n* = 10), also reporting excellent reliability [32].

Regarding the mean distribution of echo-types in our symptomatic population, we found 47.6% echo-type I, 20.3% echo-type II, 19.4% echo-type III, 12.7% echo-type IV, 67.9% echo-types I + II (aligned fibrillar structure), and 31.9% echo-types III + IV (disorganized structure) (Table 2). Elgart et al. [29] recently published normative data for Achilles tendon structure in asymptomatic individuals, stratified by age, race, and gender. They found no statistically significant differences between their age groups. For males they reported a distribution of 68.0% echo-type I, 29.5% echo-type II, 1.8% echo-type III, and 0.7% echo-type IV. Comparing these values to our findings, aligned fibrillar structure appears to be lower in our symptomatic population (67.9%) in contrast to asymptomatic peers (97.5%), while the amount of disorganized tendon structure was much higher in our study (32.1%) than reported in asymptomatic tendons (2.5%) [29].

The tendon structure of our population is quite comparable to the symptomatic population of de Jonge et al. [16], who evaluated the midportion Achilles tendon structure in nonoperatively treated mid-AT. The echo-type distribution in their study was 48.6% echo-type I, 26.0% echo-type II, 14.3% echo-type III, 11.1% echo-type IV, 74.6% echo-types I + II, and 25.4% echo-types III + IV. Comparing the aligned fibrillar structure of our study to the population of de Jonge et al. [16], we find that the values for echo-type I were 47.6% and 48.6%, and for echo-type II 20.3% and 26.0%, respectively. While echo-type I appears to be equally distributed between both symptomatic populations, echo-type II was lower in our study. This may be due to a very low representation of female participants in our population (Table 1), as it has been shown that asymptomatic female Achilles tendons contain more echo-type II in both the insertion and the midportion, compared to male tendons [32].

We tried to avoid several sources of bias that could have distorted the results of our study. To prevent review bias, all UTC scans were anonymized and independently processed by the two raters. The first rater (MM) processed each UTC scan twice to determine the intra-rater reliability. These ratings were performed with at least 4 weeks in between, in order to prevent recall bias. In general, the most recommended interval between tests during test-retest reliability assessment is 2 weeks [33]. As the construct of a UTC scan does not change over time, with an additional 2 weeks, we were on the safe side. Furthermore, we aimed to prevent observer bias [34] due to variation in experience level of the raters, by selecting two equally experienced musculoskeletal sonographers to rate the UTC scans. In retrospect, we do not believe that observer bias plays a major role; processing UTC scans appeared to be relatively easy to learn after following a user course from the manufacturer.

Our study has several limitations. First, variation in the outcomes between the two raters in quantifying the midportion Achilles tendon structure is attributed to marking different tendon boundaries, while variation in the AoMD was due to selecting a different slide considered to have the lowest representation of echo-type I. We did not explore the nature and extent of these variations, as our primary interest was to determine the intra-rater and inter-rater reliability of the quantitative analyses for Achilles tendon structure. Scanning tendon cross-sectional area using ultrasonography has proved to be less accurate than MRI, both in the Achilles tendon and patellar tendon [35]. Therefore, contour marking is a major influencer, especially when one expresses the different echo-types as percentages.

Second, with respect to the processing of scans, we have chosen to standardize the Achilles tendon midportion based on symptom location (2–7 cm proximal to the calcaneus), analogous to the generally accepted definition of mid-AT in clinical practice and scientific research [22]. This choice was made as uniformity in clinical terminology may contribute to accurate diagnostics, effective treatment, and targeted research. While processing the UTC scans in our study, we have noticed that resting Achilles tendon lengths can vary largely between subjects. Despite these anatomical variations, our midportion standardization has been applicable to the processing of all scans in our study. However, it is possible that our standardization is not applicable to all individuals, especially to those with a free tendon shorter than 2 centimeters. For this reason, future studies may consider analyzing a region of interest that defines the Achilles tendon midportion, as previously conducted in UTC research [14,16].

Third, our ICCs may be generalized to diagnosing patients suffering from mid-AT, or to the evaluation of interventions targeting the Achilles tendon structure in these individuals. However, it should be acknowledged that our results are based on a single UTC scan. In clinical practice, physical data acquisition over time, combined with short-term variations in tendon architecture, may introduce additional unexplained variability. Moreover, our ICCs may be of limited generalizability to the assessment of asymptomatic Achilles tendons, as UTC is used to evaluate load [19,20] or sometimes to predict injury [36] in asymptomatic subjects. Finally, our results may also be of limited generalizability to the female population, since only 2 out of 50 service members were female.

## 5. Perspective

UTC is an imaging modality that can visualize the Achilles tendon structure and quantify the Achilles tendon matrix integrity [14]. Normative data for tendon structure have been recently published [29,32].

In asymptomatic individuals, UTC is used to monitor load [19,20] and predict injury [36,37], while in clinical practice it can be used to establish a diagnosis of AT or to evaluate interventions targeting Achilles tendons structure [15–18].

The intra-rater and inter-rater reliability of the processing of UTC scans for Achilles tendon structure have not previously been investigated in a large cohort of subjects with mid-AT [21]. Although our ICCs show overall excellent reliability, it should be emphasized that the corresponding MDCs have to be taken into account when evaluating tendon structure in mid-AT. In general, our MDCs for midportion tendon structure are relatively low, while in the AoMD they reach up to 10%.

Growing evidence indicates that UTC of Achilles tendon structure should not be used as a biomarker for explaining the presence or severity of current and future symptoms [16,37]; however, there is conflicting evidence on this topic [14,36]. In a future study, we aim to determine if, and to what extent, our midportion structural assessment and the AoMD are associated with self-perceived pain and function by means of the VISA-A questionnaire [38].

## Figures and Tables

**Table 1:** Patient characteristics.

Measure	Total group (*n *=* *50) mean ± SD
Age (years)	41.1 ± 9.9
Height (cm)	185.0 ± 6.8
Weight (kilograms)	93.1 ± 14.9
Body mass index (%)	27.2 ± 3.5
Gender (male/female)	48/2
Duration of symptoms (months)	15.0 ± 22.3
Baseline VISA-A	59.8 ± 17.0
Numeric rating scale for maximum pain (0–10)	6.5 ± 1.6

SD: standard deviation.

**Table 2:** Mean echo-types percentages of the participants' Achilles tendons (reviewer 1, MM; rating 1).

Echo-type	Midportion total group (*n *=* *50) mean ± SD (min–max)	AoMD total group (*n *=* *50) mean ± SD (min–max)
Echo-type I%	47.6 ± 11.9 (24.7–75.2)	37,1 ± 12,2 (14.7–64.8)
Echo-type II%	20.3 ± 5.3 (10.5–35.8)	22.5 ± 7.6 (10.8–42.9)
Echo-type III%	19.4 ± 9.2 (2.6–43.0)	26.6 ± 12.2 (1.5–49.7)
Echo-type IV%	12.7 ± 6.7 (1.9–29.2)	13.5 ± 6.8 (0.4–28.0)
Total	100%	100%
Echo-type I + II%	67.9 ± 15.0 (40.4–95.3)	59.6 ± 17.6 (30.0–98.1)
Echo-type III + IV%	31.9 ± 15.3 (4.7–59.6)	40.1 ± 18.0 (1.9–70.0)
Total	100%	100%

Echo-types I, II, III, and IV are expressed as a percentage of the analyzed Achilles tendon volume. Combined, the echo-types I + II represent aligned fibrillar structure, and the echo-types III + IV disorganized tendon structure. SD: standard deviation; min: minimum; max: maximum; AoMD: area of maximum degeneration (1 slide in the Achilles tendon midportion with the lowest representation of echo-type I).

**Table 3:** Intra-rater and inter-rater reliability for the processing of UTC scans in quantifying the structure of the Achilles tendon midportion (*n *=* *50).

Echo-type	Intra-rater reliability			Inter-rater reliability		
	ICC (95% CI)	SEM	MDC	ICC (95% CI)	SEM	MDC
Echo-type I%	0.98 (0.97–0.99)	1.7	4.7	0.99 (0.95–0.99)	1.2	3.3
Echo-type II%	0.97 (0.94–0.99)	0.9	2.5	0.98 (0.96–0.99)	0.7	2.1
Echo-type III%	0.98 (0.96–0.99)	1.3	3.6	0.99 (0.95–0.99)	0.9	2.6
Echo-type IV%	0.99 (0.97–0.99)	0.7	1.9	0.99 (0.99–1.00)	0.7	1.9
Echo-type I + II%	0.98 (0.96–0.99)	2.1	5.9	0.99 (0.97–1.00)	1.5	4.2
Echo-type III + IV%	0.99 (0.97–0.99)	1.5	4.2	0.98 (0.95–0.99)	2.2	6.0

Intra-rater and inter-rater reliability are calculated for echo-types I, II, III, and IV individually, and combined for aligned fibrillar structure (echo-types I + II) and disorganized tendon structure (echo-types III + IV). CI: confidence interval; ICC: intraclass correlation coefficient; MDC: minimal detectable change (%); SEM: standard error of measurement (%).

**Table 4:** Intra-rater and inter-rater reliability for the processing of UTC scans in quantifying the AoMD (*n *=* *50).

Echo-type	Intra-rater reliability			Inter-rater reliability		
	ICC (95% CI)	SEM	MDC	ICC (95% CI)	SEM	MDC
Echo-type I%	0.98 (0.97–0.99)	1.7	4.8	0.97 (0.92–0.98)	2.1	5.9
Echo-type II%	0.85 (0.75–0.91)	2.9	8.2	0.92 (0.86–0.95)	2.1	5.9
Echo-type III%	0.96 (0.94–0.98)	2.4	6.8	0.96 (0.92–0.98)	2.4	6.8
Echo-type IV%	0.94 (0.90–0.97)	1.7	4.6	0.97 (0.95–0.99)	1.2	3.3
Echo-type I + II%	0.96 (0.93–0.98)	3.5	9.8	0.98 (0.95–0.99)	2.5	6.9
Echo-type III + IV%	0.96 (0.93–0.98)	3.6	10.0	0.97 (0.94–0.98)	3.1	8.6

Intra-rater and inter-rater reliability are calculated for echo-types I, II, III, and IV individually, and combined for aligned fibrillar structure (echo-types I + II) and disorganized tendon structure (echo-types III + IV). CI: confidence interval: ICC: intraclass correlation coefficient: MDC: minimal detectable change (%); SEM: standard error of measurement (%); AoMD, area of maximum degeneration (1 slide in the Achilles tendon midportion with the lowest representation of echo-type I).

## Data Availability

The data supporting the findings of this study are available from the corresponding author upon reasonable request.
